# Berberine Is a Novel Type Efflux Inhibitor Which Attenuates the MexXY-Mediated Aminoglycoside Resistance in *Pseudomonas aeruginosa*

**DOI:** 10.3389/fmicb.2016.01223

**Published:** 2016-08-05

**Authors:** Yuji Morita, Ken-ichi Nakashima, Kunihiko Nishino, Kenta Kotani, Junko Tomida, Makoto Inoue, Yoshiaki Kawamura

**Affiliations:** ^1^Department of Microbiology, School of Pharmacy, Aichi Gakuin UniversityNagoya, Japan; ^2^Laboratory of Medicinal Resources, School of Pharmacy, Aichi Gakuin UniversityNagoya, Japan; ^3^Department of Biomolecular Science and Regulation, Institute of Scientific and Industrial Research, Osaka UniversityOsaka, Japan

**Keywords:** *Pseudomonas aeruginosa*, efflux, *mexXY*, aminoglycoside resistance, berberine

## Abstract

The emergence and spread of multidrug-resistant *P. aeruginosa* infections is of great concern, as very few agents are effective against strains of this species. Methanolic extracts from the Coptidis Rhizoma (the rhizomes of *Coptis japonica* var. *major Satake*) or Phellodendri Cortex (the bark of *Phellodendron chinense* Schneider) markedly reduced resistance to anti-pseudomonal aminoglycosides (e.g., amikacin) in multidrug-resistant *P. aeruginosa* strains. Berberine, the most abundant benzylisoquinoline alkaloid in the two extracts, reduced aminoglycoside resistance of *P. aeruginosa* via a mechanism that required the MexXY multidrug efflux system; berberine also reduced aminoglycoside MICs in *Achromobacter xylosoxidans* and *Burkholderia cepacia*, two species that harbor intrinsic multidrug efflux systems very similar to the MexXY. Furthermore this compound inhibited MexXY-dependent antibiotic resistance of other classes including cephalosporins (cefepime), macrolides (erythromycin), and lincosamides (lincomycin) demonstrated using a pseudomonad lacking the four other major Mex pumps. Although phenylalanine-arginine beta-naphthylamide (PAβN), a well-known efflux inhibitor, antagonized aminoglycoside in a MexXY-dependent manner, a lower concentration of berberine was sufficient to reduce amikacin resistance of *P. aeruginosa* in the presence of PAβN. Moreover, berberine enhanced the synergistic effects of amikacin and piperacillin (and vice versa) in multidrug-resistant *P. aeruginosa* strains. Thus, berberine appears to be a novel type inhibitor of the MexXY-dependent aminoglycoside efflux in *P. aeruginosa*. As aminoglycosides are molecules of choice to treat severe infections the clinical impact is potentially important.

## Introduction

*Pseudomonas aeruginosa* is a metabolically versatile bacterium that can cause a wide range of severe opportunistic infections in patients with serious underlying medical conditions (Gellatly and Hancock, 2013). Infections caused by *P. aeruginosa* often are hard to treat; inappropriate chemotherapy readily selects multidrug-resistant *P. aeruginosa* against which very few agents are effective (Poole, 2011; Morita et al., 2014). This so-called “antibiotic resistance crisis” has been compounded by the lag in antibiotic discovery and development programs in recent years, and is jeopardizing the essential role played by antibiotics in current medical practices (Rossolini et al., 2014). Moreover, *P. aeruginosa* possesses an intrinsic resistance to many antimicrobials because of the bacterium's outer-membrane barrier, the presence of multidrug efflux transporters, and endogenous antimicrobial inactivation (Poole, 2011; Morita et al., 2015b). Although, anti-pseudomonal agents (e.g., carbapenems) have been discovered and developed, *P. aeruginosa* readily acquires resistance to individual agents via chromosomal mutations and lateral gene transfer (Poole, 2011; Morita et al., 2015b).

The resistance-nodulation-division (RND) efflux pumps play a major role in multidrug resistant phenotype attributed to both acquired and intrinsic mechanisms of resistance in *P. aeruginosa* (Poole, 2011, 2013). This pathogen expresses several three-component RND-type multidrug efflux systems, among which four, MexAB-OprM, MexCD-OprJ, MexEF-OprN, and MexXY-OprM (OprA), are reported to be significant determinants of multidrug resistance in lab and clinical isolates (Poole, 2013; Li et al., 2015). Unlike MexAB-OprM and MexXY-OprM, which contribute to intrinsic resistance, the MexEF-OprN and MexCD-OprJ systems are typically quiescent in wild-type cells (Poole, 2013). The tripartite structure consists of an integral membrane efflux transporter with broad substrate specificity (MexB, MexD, MexF, MexY), an outer membrane channel (OprM, OprJ, OprN, OprA), and a periplasmic protein adapter (MexA, MexC, MexE, MexX) (Li et al., 2015). The RND pumps use the proton motive force to capture antimicrobials from the periplasmic space and directly to extrude antimicrobials out of the cell (Li et al., 2015). Among them, the MexXY system is the only significant determinant of efflux-mediated aminoglycoside resistance in *P. aeruginosa* (Morita et al., 2012a).

The phenyl-arginine-β-naphthylamide (PAβN, MC-207,110) is well-known as a non-specific inhibitor against the RND-type multidrug efflux pumps in *P. aeruginosa* (Lomovskaya et al., 2001). However it antagonized the anti-pseudomonas activity of aminoglycosides (amikacin and netilmicin) in a MexXY-dependent manner even though it inhibited MexXY-dependent fluoroquinolone (levofloxacin) resistance of *P. aeruginosa* (Mao et al., 2001). MP 601384, the only inhibitor of the MexXY-mediated aminoglycoside resistance has been reported to date (Jassem et al., 2011) although its chemical structure was not revealed.

In the current study we identify berberine, a natural isoquinoline alkaloid produced by a variety of plant species (Tillhon et al., 2012), potentiates aminoglycoside activity against *P. aeruginosa* including multidrug resistant isolates through screening from traditional Japanese herbal preparations, frequently prescribed as Kampo prescriptions (Watanabe et al., 2011). This compound has been reported as a potent efflux inhibitor against Gram-positive bacteria such as *Staphylococcus aureus* (Tillhon et al., 2012) but it has a weak activity against Gram-negative bacteria such as *P. aeruginosa* in part due to a substrate of multidrug efflux pumps (Tegos et al., 2002).

## Materials and methods

### Bacterial strains, plasmids, and growth conditions

The bacterial strains and plasmids used in this study are listed in Table 1. Of note various *P. aeruginosa* strains in which contribution of the MexXY efflux system to aminoglycoside resistance were assessed before (Morita et al., 2012a) are used in the main (PAO1; a reference strain, PAGU 1498 and PAGU 1606; multidrug resistant clinical isolates, PAGU 1569; pan-aminoglycoside clinical isolates) (Table 1). Among the four strains PAGU 1498 is the *agrZ-*type MexXY-overproducing mutant (Morita et al., 2012a).

**Table 1:** **Bacterial strains and plasmids**.

**Strain names**	**Other strain names**	**Relevant characteristics**	**Reference or Source**
***E. COLI***			
PAGU^g^0121	DH5α	For recombinant DNA manipulation	Morita et al., 2010
PAGU^g^0856	S17-1	For conjugational transfer	Morita et al., 2006
***P. AERUGINOSA***			
PAGU 0974	K767, PAO1	PAO1 (Poole Lab), wild type	Morita et al., 2006
PAGU^g^0975	K1525	K767 Δ*mexXY*	Sobel et al., 2003
PAGU 1498	PA7	Multidrug-resistant clinical isolate	Roy et al., 2010
		*mexXY-oprA* overexpressed	Morita et al., 2012a
PAGU^g^1565		PA7 Δ*mexXY-oprA*	Morita et al., 2012a
PAGU 1606	NCGM2. S1, IMCJ2.S1	Multidrug-resistant clinical isolate	Sekiguchi et al., 2005
PAGU^g^1659		PAGU 1606 Δ*mexXY*	Morita et al., 2012a
PAGU 1569	K2162	Pan-aminoglycoside-resistant clinical isolate	Sobel et al., 2003
PAGU^g^1627	K2170	K2162 Δ*mexXY*	Sobel et al., 2003
PAGU 0847	PAO1	PAO1 (Tsuchiya Lab), wild type	Morita et al., 2001a
PAGU^g^0849	YM34	PAGU 0847 *mexAB*::*FRT, mexCD-oprJ::FRT, mexEF-oprN::FRT*	Morita et al., 2001b
PAGU^g^0850	YM44	PAGU 0847 *mexAB*::*FRT, mexCD-oprJ*::*FRT, mexEF-oprN*::*FRT, mexXY*::*FRT*	Li et al., 2003
PAGU^g^1881		YM34 Δ*mexZ*	This study
PAGU^g^1927		YM34 Δ*mexZ, mexVW*::*gfp*-*aacC1*	This study
PAGU^g^1929		YM34 Δ*mexZ*, Δ*mexVW*	This study
PAGU^g^1931		PAGU^g^1927::Δ*mexXY*	This study
PAGU^g^1933		PAGU^g^1929::Δ*mexXY*	This study
PAGU 1607	NCGM798, IMCJ798	Multidrug-resistant clinical isolate	Kitao et al., 2009
PAGU 1608	NCGM799, IMCJ799	Multidrug-resistant clinical isolate	Kitao et al., 2009
PAGU 1640	PA9085CB	Multidrug-resistant clinical isolate	Dr. Tam's Gift, (Houston, USA)
PAGU 1675	U33b	Multidrug-resistant clinical isolate	Bradbury et al., 2010
PAGU 0217	GTC 2017, 10-49	Multidrug-resistant clinical isolate	Dr. Sawamura's Gift (Gifu, Japan)
PAGU 1249	No.514	Multidrug-resistant clinical isolate	Tsuchimochi et al., 2008
PAGU 1717	NCGM1179	Multidrug-resistant clinical isolate	Tada et al., 2011
**OTHER BACTERIA**			
PAGU 0002^T^	ATCC 27061^T^	*Achromobacter xylosoxidans* subsp. *xylosoxidans*	Yabuuchi et al., 1998
PAGU 0013^T^	ATCC 25416^T^	*Burkholderia cepacia*	Yabuuchi et al., 1992
PAGU 1567^T^	ATCC 19606^T^	*Acinetobacter baumannii*	Kumar et al., 2010
**PLASMIDS**			
pEX18Tc			Hoang et al., 1998
pCSV05-01		pEX18Tc::Δ*mexXY*	Sobel et al., 2003
pYM021		pEX18Tc::Δ*mexZ*	Morita et al., 2006
pYM145		pEX18Tc::Δ*mexVW*	This study
pPS858		Source of *aacC1*-*gfp* fragment flanked by *FRT* sites	Hoang et al., 1998
pYM146		pYM145 inserted with *aacC1-gfp* flanked by *FRT* sites	This study
pFLP2		Flp recombinase plasmid	Hoang et al., 1998

Bacterial cells were grown (unless otherwise indicated) in Luria (L) broth and on L agar (1.5%) under aerobic conditions at 37°C, as previously described, with antibiotics as specified (Morita et al., 2015b). Bacterial growth was quantified by measuring the optical density at 600 nm on an Ultrospec 2100 Pro Spectrophotometer (GE Healthcare Corp., Tokyo, Japan), unless otherwise indicated. Cells harboring the plasmid pEX18Tc (Hoang et al., 1998) or derivatives thereof were maintained on medium supplemented with 2.5–10 μg/ml tetracycline for *E. coli* or and selected on medium supplemented with 20–150 μg/ml tetracycline for *P. aeruginosa*. Cells harboring the plasmid pFLP2 (Hoang et al., 1998) were maintained and selected on medium supplemented with 100 μg/ml ampicillin for *E. coli* or 50–200 μg/ml carbenicillin for *P. aeruginosa*.

### Construction of *P. aeruginosa* mutants

In-frame deletion mutants and/or *aacC1-gfp* insertion mutants of *mexXY, mexZ*, and *mexVW* from *P. aeruginosa* PAO1 derivatives were constructed using the previously described *sacB*-based strategy (Morita et al., 2006, 2010, 2015a). The plasmids and resulting *P. aeruginosa* mutants are listed in Table 1, while the primer pairs are listed in Table 2. To introduce gene deletions into strains of *P. aeruginosa*, deletion constructs were first prepared in plasmid pEX18Tc by cloning PCR-amplified 0.75-kb DNA fragments corresponding to the regions upstream and downstream of the gene sequences to be deleted. The selection concentrations of tetracycline and gentamicin for the recombination events were adjusted to reflect the endogenous tetracycline and gentamicin MICs of the *P. aeruginosa* strains. These constructs were confirmed by colony PCR.

**Table 2:** **Primers used in this study**.

**Primer**	**Sequence (5′–3′)**	**Purpose**	**References**
SacI*-mexVW*-UF	GCTAGAGCTCCTGGTAGTGGCCAACGGCG	*mexVW* genes disruption of *P. aeruginosa* PAO1 derivatives	This study
BamHI-*mexVW*-UR	CTGAGGATCCCATAATCCTGGTCCCTGGTATGCC	*mexVW* genes disruption of *P. aeruginosa* PAO1 derivatives	This study
BamHI-*mexVW*-DF	GCATGGATCCTGATCGGAAACGGCGGAC	*mexVW* genes disruption of *P. aeruginosa* PAO1 derivatives	This study
HindIII-*mexVW*-DR	CTAGAAGCTTTGCCGAGGGGCTTGAGGT	*mexVW* genes disruption of *P. aeruginosa* PAO1 derivatives	This study

### Preparation of crude drug extracts

A total of 96 crude drugs (listed in Table S1) that are used in Kampo prescriptions in Japan (Tanabe et al., 2014) were prepared as follows. Ten grams of each crude drug was placed into 100 ml of methanol and extracted for 1 day at room temperature, and then the solution was filtered. The extraction process was performed three times. Each filtrate was mixed and concentrated *in vacuo* at 40°C by using a rotary evaporator N-1000 (EYELA, Tokyo, Japan) equipped with a coolant system CCA-1110 (EYELA, Tokyo, Japan). Each final extract was dissolved in dimethyl sulfoxide (DMSO) and adjusted to the concentration of 100 mg/ml. Each of the crude drugs was resuspended at 1 mg/ml in DMSO, and evaluated on the condition of the broth micro-dilution method described above at the Section of Preparation of Crude Drug Extracts for the ability to restore effectiveness of either 16 μg/ml imipenem, 4 μg/ml ciprofloxacin, or 32 μg/ml amikacin against highly multidrug-resistant *P. aeruginosa* PAGU 1606.

### Antibiotic susceptibility assay

The susceptibility of *P. aeruginosa* to antimicrobial agents in cation-adjusted Mueller–Hinton broth was assessed using the two-fold serial micro-titer broth dilution method described previously (Morita et al., 2012a). Minimal inhibitory concentrations (MICs) were defined as the lowest concentration of antibiotic resulting in visible inhibition of growth after about 18–22 h of incubation at 37°C (for *P. aeruginosa*) or after about 20–24 h of incubation at 35°C (for *Achromobacter xylosoxidans, Burkholderia cepacia*, and *Acinetobacter baumannii*). The categorization as susceptible, intermediate, and resistant was performed according to the interpretive standards of the Clinical and Laboratory Standards Institute (CLSI).

The fractional inhibitory concentration (FIC) index was calculated as reported elsewhere (Lomovskaya et al., 2001). The effects of the drugs were interpreted to be indicative of synergy when the index was ≤ 0.5.

Amikacin, ampicillin, azithromycin, berberine, carbenicillin, ciprofloxacin, erythromycin, gentamicin, lincomycin, and tetracycline were purchased from Wako Pure Chemicals Industries, Ltd. (Osaka, Japan). Phenylalanine-arginine β-naphthylamide was purchased from Sigma-Aldrich Co. LLC (Tokyo, Japan). Imipenem/cilastatin and cefepime were purchased from Sandoz K.K. (Tokyo, Japan). Piperacillin was purchased from Nichi-Iko Pharmaceutical Co., Ltd. (Toyama, Japan). Tobramycin was purchased from Towa Pharmaceutical Co., Ltd. (Kadama, Osaka, Japan). Arbekacin was purchased from Shiono Chemical Co., Ltd. (Fujioka, Gunma, Japan).

### High performance liquid chromatographic method for quantification of berberine and coptisine

The stock solutions of berberine and coptisine were both prepared in methanol. The extracts also were dissolved in methanol to a concentration of 1 mg/ml. In order to construct calibration curves, a series of standard solution of each compound was prepared by appropriate dilution of the stock solutions. Calibration curves were constructed by plotting the peak area ratio vs. the concentration.

The high performance liquid chromatographic conditions were as follows: pump, Hitachi L-2130; autosampler, Hitachi L-2200; detector, Hitachi L-2455; column, YMC Pack ODS-AP302 (4.6 x 150 mm, YMC); mobile phase, 0.1 M phosphate buffer (pH 2.1)—methanol (70:30, v/v); flow rate, 1 ml/min; column temperature, 25°C; DAD, 340 nm; and injection volume, 10 μl.

### Molecular biology techniques

Plasmid DNA isolation from *E. coli*, DNA purification, measurement of DNA concentration, DNA digestion with restriction enzymes, DNA dephosphorylation, DNA ligation, isolation of chromosomal DNA from *P. aeruginosa*, PCR conditions, nucleotide sequencing, competent cell preparation from *E. coli*, transformation of *E. coli*, and transfer of plasmids into *P. aeruginosa* via conjugation were performed as described previously (Morita et al., 2015b), unless otherwise indicated. DNA sequences and amino acid sequences were analyzed using the Pseudomonas Genome Database (Winsor et al., 2011), Basic Local Alignment Search Tool (BLAST), and the software DNASIS Pro (Ver. 2.1; Hitachi, Japan).

## Results

### Isolation of a novel inhibitor of the MexXY multidrug efflux system from the rhizomes of *Coptis Japonica* and the bark of *Phellodendron Amurense*

Extracts from the Coptidis Rhizoma (the rhizomes of *Coptis japonica* var. *major Satake*) or Phellodendri Cortex (the bark of *Phellodendron chinense* Schneider) restored the effectiveness of 32 μg/ml amikacin against the highly multidrug-resistant *P. aeruginosa* PAGU 1606. None of the tested extracts restored susceptibility of the strain to 16 μg/ml imipenem or 4 μg/ml ciprofloxacin (data not shown). The extracts of both *Coptis* rhizome and *Phellodendron* bark reduced MICs of amikacin and gentamicin (but not those of imipenem or ciprofloxacin) by eight-fold or more in the two multidrug resistant *P. aeruginosa* strains (PAGU 1606 and PAGU 1498) (Table 3).

**Table 3:** **Methanol extracts from the Coptidis Rhizoma restore aminoglaycoside susceptibility in multidrug-resistant ***P. aeruginosa*****.

***P. aeruginosa* Strain**	**Relevant property**	MIC (μg/ml)^*^									
		**AMK**		**GEN**		**CIP**		**FEP**		**IPM**	
		**−**	**+**	**−**	**+**	**−**	**+**	**−**	**+**	**−**	**+**
PAGU 1606	Multidrug resistant	256	32	64	8	64	64	>128	>128	128	128
PAGU 1498	Multidrug resistant	32	4	>512	64	128	64	32	16	1	2
PAO1 (PAGU 0974)	Wild type	2	1	2	0.5	0.25	0.25	4	4	0.5	1

*AMK, amikacin; GEN, gentamicin; CIP, ciprofloxacin; FEP, cefepime; IPM, imipenem*.

* *MICs are measured in the absence (−) or presence (+) of methanol extract from the Coptidis Rhizoma*.

### Berberine is a novel inhibitor of aminoglycoside resistance dependent on the MexXY-efflux system in *P. aeruginosa*

Berberine is reported the most abundant benzylisoquinoline alkaloid in the two active extracts (Tillhon et al., 2012). Therefore, we next tested the activity of berberine along with coptisine, a similar benzylisoquinoline alkaloid. Berberine constituted 34.4% of the extract from the rhizomes of *C. japonica* and 28.3% of extract from the bark of *Phellodendron amurense* (Figure S1). Coptisine was the second-most abundant benzylisoquinoline alkaloid from the *Coptis* extract, constituting 4.1% of the extract from the rhizomes of *C. japonica* (Figure S1). Although berberine was ineffective as a solo antibacterial (with an MIC >512 μg/ml MIC against *P. aeruginosa*; data not shown), berberine inhibited aminoglycoside resistance by two- to >eight-fold against four *P. aeruginosa* strains (Table 4). Such a reduction was not observed in isogenic mutants deficient in the RND-type multidrug efflux system MexXY, which is considered one of the major aminoglycoside resistance determinants in *P. aeruginosa* (Morita et al., 2012b) (Table 4). Coptisine showed activity similar to that of berberine (data not shown). These results indicate that the inhibitory activity of the two extracts reflected, at least in part, the activity of berberine and/or coptisine. Of note berberine also inhibited aminoglycoside resistance of *A. xylosoxidans* PAGU 0002^T^ and *B. cepacia* PAGU 0013^T^ but not that of *A. baumannii* PAGU 1567^T^ (Table 5).

**Table 4:** **Berberine attenuates aminoglycoside resistance of ***P. aeruginosa*** in the MexXY -dependent manner**.

**Strains**	**Relevant property**	**MIC (μg/ml) with (+) and without (–) addition of 256 μg/ml BB**							
		**AMK**		**ABK**		**GEN**		**TOB**	
		**−**	**+**	**−**	**+**	**−**	**+**	**−**	**+**
PAGU 1606	Multidrug resistant	256	32	32	4	64	16	256	64
PAGU^g^1659	PAGU 1606 Δ*XY*	8	4	1	0.5	1	0.5	16	8
PAGU 1498	Multidrug resistant	32	4	32	4	>512	128	>256	32
PAGU^g^1565	PAGU 1498 Δ*XY*	2	1	2	1	16	8	16	8
PAGU 1569	Pan-AG resistant	256	64	128	16	256	64	32	16
PAGU^g^1627	PAGU 1569 Δ*XY*	32	16	16	16	8	8	4	4
PAGU 0974	Wild type, PAO1	2	1	1	0.5	2	1	0.5	0.25
PAGU^g^0975	PAGU 0974 Δ*XY*	0.5	0.5	0.25	0.25	0.25	0.25	0.25	0.125

*AG, aminoglycoside; AMK, amikacin; ABK, arbekacin; BB, berberine; GEN, gentamicin; TOB, tobramycin; XY, mexXY*.

**Table 5:** **Berberine attenuates aminoglycoside resistance of ***A. xylosoxidans*** and ***B. cepacia*****.

**Strains**	**MIC (μg/ml) with (+) and without (–) addition of 256 μg/ml BB**							
	**AMK**		**ABK**		**GEN**		**TOB**	
	**–**	**+**	**–**	**+**	**–**	**+**	**–**	**+**
*A. xylosoxidans* PAGU 0002^T^	>512	64	>512	64	>512	16	512	16
*B. cepacia* PAGU 0013^T^	256	16	128	16	256	16	256	8
*A. baumannii* PAGU 1567^T^	32	32	32	32	64	64	8	8

*AMK, amikacin; ABK, arbekacin; BB, berberine; GEN, gentamicin; TOB, tobramycin*.

### Berberine inhibits MexXY-mediated resistance to erythromycin, lincomycin, and cefpirome in a *P. aeruginosa* mutant lacking four RND multidrug efflux systems (MexAB, MexCD, MexEF, and MexVW)

To test the effect of berberine on non-aminoglycoside susceptibility, we, first of all, evaluated MICs against the wild-type strain (PAO1) and then, in order to remove any effects of the RND pumps, evaluated MICs in a multidrug-sensitive *P. aeruginosa* strain mutated in the loci encoding four RND multidrug efflux systems (*mexXY, mexAB, mexCD-oprJ*, and *mexEF-oprN*) (Morita et al., 2001b; Li et al., 2003) (Table 6). Like the parent strain, the quadruple efflux mutant (PAGU^g^ 0850) also exhibited an eight-fold decrease in MIC of erythromycin in the presence of berberine (Table 6). However mutation of one of the remaining RND multidrug efflux systems (*mexVW*) attenuated the effect of berberine, with the quintuple mutant (PAGU^g^ 1933) exhibiting only a two-fold decrease in erythromycin MIC upon exposure to berberine (Table 6). Similar effects were seen for lincomycin resistance (Table 6). These results suggest that berberine attenuation of resistance to the macrolides and lincomycin depends on the MexVW multidrug efflux system. The molecular mechanism underlying the berberine effects on the MexVW system will be the subject of further research.

**Table 6:** **Berberine inhibits MexXY- or MexVW-mediated resistance of ***P. aeruginosa*** mutants**.

**Strain**	**Relevant property**	*mex* gene^*^ present	**MIC (μg/ml) with (+) and without (–) addition of 256 μg/ml BB**									
			**ERY**		**LIN**		**FEP**		**CIP**		**TET**	
			**−**	**+**	**−**	**+**	**−**	**+**	**−**	**+**	**−**	**+**
PAGU 0974	Wild type, PAO1	*ABM* *CDJ* *EFN* *XY* *VW*	256	64	>4096	4096	nd	nd	0.25	0.25	32	16
PAGU^g^0850	Δ*AB* Δ*CDJ* Δ*EFN* Δ*XY*	*M* *VW*	64	8	1024	16	nd	nd	nd	nd	nd	nd
PAGU^g^1929	Δ*AB* Δ*CDJ* Δ*EFN* Δ*VW*	*M* *XY*	256	32	2048	256	2	0.25	0.125	0.015	4	0.5
PAGU^g^1933	Δ*AB* Δ*CDJ* Δ*EFN* Δ*XY* Δ*VW*	*M*	8	4	16	8	0.125	0.125	0.008	0.002	0.03	0.008

* *mex genes here mean mexAB-oprM, mexCD-oprJ, mexEF-oprN, mexXY, and mexVW*.

*BB, berberine; ERY, erythromycin; LIN, lincomycin; FEP, cefepime; CIP, ciprofloxacin; TET, tetracycline; ABM, mexAB-oprM; CDJ, mexCD-oprJ; EFN, mexEF-oprN; XY, mexXY; VW, mexVW; AB, mexAB; M, oprM; nd, not done*.

We sought to better understand the molecular mechanism of berberine attenuation of the MexXY-mediated efflux. Therefore, adjuvant effects of berberine on resistance to various antimicrobial substrates of the MexXY efflux pump were compared in two RND mutant strains differing only by the absence or presence of *mexXY* (PAGU^g^ 1929 vs PAGU^g^ 1933, respectively; Table 6). Specifically, the *mexXY* genes were overexpressed in PAGU^g^ 1933 due to deletion of the *mexZ* repressor gene. The effects of *mexXY* overexpression were being determined in the absence of other major RND multidrug efflux systems, since the two strains shared deletions in the *mexAB, mexCD-oprJ, mexEF-oprN*, and *mexVW* loci. As seen in the earlier experiments (e.g., Table 6), resistance to erythromycin, lincomycin, cefpirome, and aminoglycosides was attenuated eight-fold in the presence of berberine, and these effects were observed only in the presence of the MexXY system (Table 6). While resistance to ciprofloxacin and tetracycline also was attenuated eight-fold in the presence of berberine, a partial berberine effect (four-fold decrease in MIC) was observed for ciprofloxacin and tetracycline even in the absence of *mexXY* overexpression (compare PAGU^g^ 1929 and PAGU^g^ 1933; Table 6). It might suggest that berberine possibly has another target sites other than 5 RND pumps which are involved in resistance to ciprofloxacin and tetracycline. In control experiments (data not shown), we confirmed that berberine did not display antimicrobial activity (at concentrations up to 512 μg/ml) in the PAGU^g^ 1929 strain (that is, despite deletion of *mexAB, mexCD-oprJ, mexEF-oprN*, and *mexVW*). We additionally confirmed that the attenuation activity of berberine in the PAGU^g^ 1929 background was still concentration-dependent; the aminoglycoside adjuvant activity of berberine in this strain was not detected at a berberine concentration of 8 μg/ml (data not shown).

### Comparison of berberine with the known efflux inhibitor phenylalanine-arginine β-naphthylamide (PAβN)

We compared the effects of berberine with those of the previously reported efflux inhibitor PAβN. Consistent with earlier reports (Mao et al., 2001), PAβN did not reduce MexXY-dependent gentamicin resistance (as seen in PAGU^g^ 1929), but PAβN did inhibit ciprofloxacin resistance in the quadruple efflux mutant (*mexAB, mexCD-oprJ, mexEF-oprN*, and *mexVW*; PAGU^g^ 1929) (Table 7). This distinction suggests that berberine inhibits the MexXY system via a mechanism distinct from that of PAβN. Given that berberine is known to be substrates of many multidrug efflux pumps, including RND-type multidrug efflux pumps (Tegos et al., 2002), PAβN is expected to facilitate accumulation of berberine in *P. aeruginosa* cells. Indeed, exposure to PAβN provided inhibition of MexXY-dependent aminoglycoside resistance at a lower concentration of berberine, as shown for the multidrug-resistant *P. aeruginosa* strain PAGU 1606 (Table S2).

**Table 7:** **PAβN inhibits MexXY-mediated resistance to ciprofloxacin but not to gentamicin, in ***P. aeruginosa*** mutants**.

**Strain**	**Relevant property**	*mex* genes^*^ present	MIC (μg/ml) with (+) and without (–) addition of PAβN^**^				
			**GEN**		**CIP**		**PAβN**
			**−**	**+**	**−**	**+**	
PAGU^g^1929	Δ*AB* Δ*CDJ* Δ*EFN* Δ*VW*	*M* *XY*	2	4	0.125	0.0078	512
PAGU^g^1933	Δ*AB* Δ*CDJ* Δ*EFN* Δ*VW* Δ*XY*	*M*	0.25	0.25	0.0078	0.0039	32

* *mex genes here mean mexAB-oprM, mexCD-oprJ, mexEF-oprN, mexXY, and mexVW*.

** *Concentrations of PAβN are 1/8-fold MICs for PAβN of PAGU^g^1929 and PAGU^g^1933, respectively*.

*GEN, gentamicin; CIP, ciprofloxacin; PAβN, phenylalanine-arginine β-naphthylamide; ABM, mexAB-oprM; CDJ, mexCD-oprJ; EFN, mexEF-oprN; XY, mexXY; VW, mexVW; AB, mexAB; M, oprM*.

### Berberine synergistically inhibits MexXY-mediated aminoglycoside resistance in *P. aeruginosa*

FIC indices were determined in multiple strains for the combination of gentamicin and berberine (Table 8). The combination exhibited synergy only in MexXY-positive strains (PAGU^g^1927 and PAGU^g^1929). This result suggests that berberine and gentamicin act synergistically on the MexXY system. Puzzlingly, berberine MICs in the presence of gentamicin are significantly higher in the MexXY-deficient *P. aeruginosa* mutant cells than in the MexXY-overproducing *P. aeruginosa* mutant cells (Table 8). This observation implies that berberine accumulates more effectively in the presence of the MexXY system, although classically the pump facilitates the *efflux* of substrates.

**Table 8:** **Berberine synergistically inhibits MexXY-mediated gentamicin resistance in ***P. aeruginosa*** mutants**.

**Strain**	***XY***	***AAC***	**MIC (μg/ml) for GEN in the presence of:**		**MIC (μg/ml) for BB in the presence of:**		**FIC**	**Mode of Interaction**
			**−**	**+BB**	**−**	**+GEN**		
PAGU^g^1927	+	+	1024	256	>512	128	<0.5	Synergy
PAGU^g^1931	−	+	8	16	>512	>512	>2.0	Indifferent
PAGU^g^1929	+	−	8	2	>512	128	<0.5	Synergy
PAGU^g^1933	−	−	0.25	0.25	>512	>512	>1.0	Indifferent

GEN, gentamicin; BB, berberine; XY, mexXY; AAC, aacC1^*^.

^*^aacC1 is a gentamycin acetyl transferase-encoding gene derived from pPS858 (Hoang et al., 1998).

### Berberine promotes combined effects of amikacin with piperacillin (and vice versa) in multidrug-resistant *P. aeruginosa* strains

Aminoglycoside uptake is known to be facilitated by inhibitors of bacterial cell wall synthesis such as β-lactams (Taber et al., 1987). Anti-pseudomonal β-lactams and polymyxins thus are expected to promote the adjuvant effects of berberine observed above. We tested several such agents (data not shown) and found that piperacillin, an anti-pseudomonal β-lactam, was among the most potent. The effects of this cell wall inhibitor were seen in all of the tested multidrug-resistant *P. aeruginosa* strains (Table 9). Notably, exposure to the combination of piperacillin (32 μg/ml) and berberine (512 μg/ml) reduced the amikacin MIC of PAGU 1606 to a level below the amikacin breakpoint of 32 μg/ml (Table 9). In contrast, exposure to piperacillin or berberine alone did not have as large an effect in reducing amikacin resistance. We additionally noted a reduction in the MIC of piperacillin in PAGU 1606 when grown in the presence of both of berberine (512 μg/ml) and sub-breakpoint amikacin (16 μg/ml), with the piperacillin MIC reduced to a level similar to that seen when grown in the presence of 128 μg/ml of amikacin alone (Table 9). Indeed, the FIC index of amikacin in combination with piperacillin of PAGU 1606 was calculated as follows: amikacin MIC in the presence of piperacillin/amikacin MIC) + (piperacillin MIC in the presence of amikacin/piperacillin MIC) = 64/256+16/256 < 0.5. It suggested synergy of these two compounds in PAGU 1606 (Table 9).

**Table 9:** **Berberine enhances synergistic effect of piperacillin and amikacin (and vice versa) in multidrug-resistant ***P. aeruginosa*** strains**.

**Strain**	**AMK MIC (μg/ml) in the presence of:**				**PIPC MIC (μg/ml) in the presence of:**			
	**−**	**BB (512)**	**PIPC (32)**	**BB (512) PIPC (32)**	**−**	**BB (512)**	**AMK (128)**	**BB (512) AMK (16)**
PAGU 1606	256	64	64	16	256	256	16	32
PAGU 1607	256	nd	nd	16	256	nd	nd	32
PAGU 1608	256	nd	nd	16	256	nd	nd	32
PAGU 1640	64	nd	nd	16	1024	nd	nd	64
PAGU 1675	>1024	nd	nd	64	1024	nd	nd	256
PAGU 0217	256	nd	nd	8	256	nd	nd	32
PAGU 1249	>1024	nd	nd	4	256	nd	nd	<0.5
PAGU 1717	128	nd	nd	2	128	nd	nd	8

*AMK, amikacin; BB, berberine, PIPC, piperacillin; nd, not done*.

*Values in parentheses are concentrations (μg/ml) of indicated drugs*.

## Discussion

The MexXY multidrug efflux system is a significant determinant of resistance to aminoglycosides in *P. aeruginosa*, although aminoglycosides are not standard RND pump substrates (Morita et al., 2012b). The MexXY pump typically is encoded by a two-gene operon and comprises a periplasmic membrane fusion protein (MexX) and an inner-membrane (IM) drug/H^+^ antiporter (MexY; the RND component); the pump is functional only when combined with an outer-membrane (OM) channel encoded by a separate locus (e.g., OprM, which is encoded by the third gene of another multidrug efflux operon, *mexAB-oprM* Morita et al., 2012b). In some *P. aeruginosa* strains, such as the taxonomic outlier strain PA7, the *mexXY* genes are adjacent to a third gene (*oprA* in PA7) encoding an outer membrane channel; the resulting three-gene operon is similar to *axyXY-oprZ* of *A. xylosoxidans* and *amrAB-oprA* of various *Burkholderia* species (e.g., *B. pseudomallei* and *B. cepacia* complexes) (Morita et al., 2012a,b). In the PA7-related strains, MexXY seem to cooperate with both OprM and OprA, as either of these Opr proteins can compensate for the genetically engineered suppression of the other (Morita et al., 2012b; Li et al., 2015). In *P. aeruginosa*, the MexXY system mediates resistance to aminoglycosides as well as various clinically relevant antimicrobials such as fluoroquinolones, some β-lactams (e.g., cefepime), tetracyclines (including tigecycline), and macrolides such as azithromycin (Morita et al., 2012b). In wild-type cells, the *mexXY* operon is induced by agents that target ribosomes (Jeannot et al., 2005; Morita et al., 2006); the operon is overexpressed in mutant cells (e.g., Morita et al., 2012b; Guénard et al., 2014). Potent inhibitors of RND-type efflux pumps could be used as adjunctive therapies that would increase the potency of existing antibiotics and decrease the emergence of multidrug resistance in Gram-negative pathogens such as *P. aeruginosa* and *Enterobacteriaceae* (Gill et al., 2015; Opperman and Nguyen, 2015; Venter et al., 2015). Several potent inhibitors of RND-type efflux pump (e.g., PAβN (Lomovskaya et al., 2001), D13-9001 (Nakashima et al., 2013), and MBX2319 Vargiu et al., 2014) have been reported. However, only a single inhibitor of MexXY (MP 601384) has been reported to date (Jassem et al., 2011); the chemical structure of that agent was not revealed.

In the present study, berberine was shown to synergistically inhibit aminoglycoside resistance in *P. aeruginosa* in a MexXY-dependent manner. We observed a similar effect in *A. xylosoxidans* and *B. cepacia*, distinct species that possess MexXY orthologues (Morita et al., 2012b). On the other hand, we did not observe berberine attenuation of aminoglycoside resistance in *A. baumannii*. In *Acinetobacter*, the AdeAB RND-type pump mediates aminoglycoside resistance (Magnet et al., 2001), and the AdeAB system exhibits more sequence similarity to *P. aeruginosa* MexCD than to *P. aeruginosa* MexXY (Morita et al., 2012b). Furthermore, berberine attenuated MexXY-dependent resistance to aminoglycosides in a *P. aeruginosa* harboring MexXY but lacking another four RND multidrug efflux systems. We observed the same effect in this background for antimicrobials of other classes (including erythromycin, cefepime, and lincomycin) that are considered substrates of the MexXY pump (Morita et al., 2012b). Berberine is a natural isoquinoline alkaloid produced by a variety of plant species; this compound has been reported to possess a number of biological activities, including antimicrobial effects (Tillhon et al., 2012). Recently, berberine's antibacterial properties were shown to be due primarily to inhibition of the cell division protein FtsZ (Domadia et al., 2008; Boberek et al., 2010). However, this compound's antibacterial activity is not strong against Gram-negative bacteria such as *P. aeruginosa* at least in part because many bacterial multidrug efflux pumps recognize berberine as a substrate (e.g., Morita et al., 1998; Tegos et al., 2002). Berberine is more potent against Gram-positive bacteria such as *Mycobacterium tuberculosis* and *S. aureus* by inhibiting MF-type multidrug efflux pumps such as NorA (Tegos et al., 2002). The MexXY system confers berberine resistance in *Escherichia coli* lacking the major RND-type mulidrug efflux system AcrAB (data not shown) as well as confers PAβN resistance in *P. aeruginosa* lacking the major RND pumps (Table 6), suggesting that each of the two agents acts as a competitive inhibitor of substrate binding and/or extrusion. However each spectrum of antimicrobial potentiation is not the same. Unfortunately, the adjuvant activity of berberine requires (in *Pseudomonas*) a relatively high (>100 μg/ml) concentration of the compound, consistent with a limited ability to inhibit the MexXY efflux. Clinical development would require optimization of the structure of berberine to provide better synergistic activity with aminoglycosides against *P. aeruginosa*. Recently a region of MexY (the substrate-specificity-determining RND component of this pump) that corresponds to a proximal binding pocket of AcrB was shown to be involved in aminoglycoside recognition and efflux (Lau et al., 2014; Li et al., 2015). This protein domain would be a good starting place for understanding details of aminoglycoside recognition and export by MexY. Although the substrate specificity of *E. coli* AcrAB-TolC (the best studied RND-type multidrug efflux pump) is extraordinarily broad, the complex does not recognize aminoglycosides as substrates (Li et al., 2015). The design and development of better MexXY inhibitors will require improved understanding of the molecular mechanisms of MexXY-mediated aminoglycoside resistance.

In conclusion berberine is the first efflux inhibitor that restores aminoglycosides activity in multidrug resistant *P. aeruginosa*. As aminoglycosides are molecules of choice to treat severe infections the clinical impact is potentially important.

## Author contributions

YM conceived and designed the experiment. YM, KeN, and KK performed the experiments. YM, KeN, KuN, KK, JT, MI, and YK analyzed the data. YM wrote the paper.

### Conflict of interest statement

The authors declare that the research was conducted in the absence of any commercial or financial relationships that could be construed as a potential conflict of interest.
