# Supplementary material for: Berberine Is a Novel Type Efflux Inhibitor Which Attenuates the MexXY-Mediated Aminoglycoside Resistance in Pseudomonas aeruginosa
Source: Front Microbiol. 2016 Aug 5;7:1223. doi: 10.3389/fmicb.2016.01223 (PMC4975076; doi:10.3389/fmicb.2016.01223)
Supplement: Supplementary file 1 [file Table1.doc]

**Supplementary Material - Berberine is a novel type efflux inhibitor which attenuates the MexXY-mediated aminoglycoside resistance in *Pseudomonas aeruginosa*.**

**Yuji Morita^1^, Ken-ichi Nakashima^2^, Kunihiko Nishino^3^, Kenta Kotani^1^, Junko Tomida^1^, Makoto Inoue^2^, Yoshiaki Kawamura^1^**

^1^Department of Microbiology, School of Pharmacy, Aichi Gakuin University, Nagoya, Aichi, Japan
^2^Laboratory of Medicinal Resources, School of Pharmacy, Aichi Gakuin University, Nagoya, Aichi, Japan
^3^ Institute of Scientific and Industrial Research, Osaka University, Osaka, Japan

*** Correspondence:** Yuji Morita, Department of Microbiology, School of Pharmacy, Aichi Gakuin University, 1-100 Kusumoto, Chikusa, Nagoya, Aichi, 464-8650, Japan.

[yujmor@dpc.agu.ac.jp](mailto:yujmor@dpc.agu.ac.jp)

| Table S1. A total of 96 crude drugs in this study | | | |
| --- | --- | --- | --- |
| Names of herbaria | Lot No. | Names of herbaria | Lot No. |
| Achyranthis Radix | 22026591 | Lilii Bulbus | 22029511 |
| Akebiae Caulis | 23006161 | Lonicerae Folium cum Caulis | 22040701 |
| Alismatis Rhizoma | 22043631 | Lycii Cortex | 23000421 |
| Alpiniae Officinari Rhizoma | 22049031 | Magnoliae Cortex | 22028611 |
| Amomi Semen | 22042491 | Magnoliae Flos | 23012431 |
| Anemarrhenae Rhizoma | 23005341 | Menthae Herba | 22044091 |
| Angelicae Dahuricae Radix | 22049651 | Mori Cortex | 22034951 |
| Angelicae Radix | 22041851 | Nelumbis Semen | 23006011 |
| Araliae Cordatae Rhizoma | 22006261 | Notopterygii Rhizoma | 22041601 |
| Arctii Fructus | 21054451 | Nupharis Rhizoma | 20009891 |
| Arecae Semen | 22031171 | Ophiopogonis Tuber | 23003151 |
| Arisaematis Tuber | 22038971 | Paeoniae Radix | 22011231 |
| Artemisiae Capillari Flos | 22031681 | Moutan Cortex | 23005351 |
| Artemisiae Folium | 22036091 | Ginseng Radix | 23018601 |
| Asiasari Radix | 22020521 | Panacis Japonici Rhizoma | 21018391 |
| Asparagi Tuber | 23011471 | Perillae Herba | 24018411 |
| Astragali Radix | 22043331 | Phellodendri Cortex | 22038081 |
| Atractylodis Lanceae Rhizoma | 23018581 | Pinelliae Tuber | 22042141 |
| Atractylodis Rhizoma | 23001771 | Plantaginis Semen | 22049181 |
| Bambusae Caulis | 22036071 | Platycodi Radix | 22040381 |
| Benincasae Semen | 21057771 | Polygalae Radix | 22000701 |
| Bupleuri Radix | 22025091 | Polygoni Multiflori Radix | 22039451 |
| Carthami Flos | 22040711 | Polyporus | 22045121 |
| Chrysanthemi Flos | 22008841 | Armeniacae Semen | 22009451 |
| Cimicifugae Rhizoma | 21017241 | Persicae Semen | 22052901 |
| Cinnamomi Cortex | 22044881 | Puerariae Radix | 22040931 |
| Aurantii Fructus Immaturus | 22043361 | Quercus Cortex | 23001531 |
| Aurantii Nobilis Pericarpium | 23002241 | Rehmanniae Radix | 22029541 |
| Clematidis Radix | 21020771 | Rhei Rhizoma | 22041591 |
| Cnidii Rhizoma | 23001371 | Saposhnikoviae Radix | 24027411 |
| Coicis Semen | 23018091 | Saussureae Radix | 23004371 |
| Coptidis Rhizoma | 22048301 | Schisandrae Fructus | 22026581 |
| Corni Fructus | 22038491 | Schizonepetae Spica | 22042881 |
| Corydalis Tuber | 22042871 | Scutellariae Radix | 22037381 |
| Cyperi Rhizoma | 22028611 | Sinomeni Caulis et Rhizoma | 23008241 |
| Dioscoreae Rhizoma | 23000221 | Sophorae Radix | 22011021 |
| Ephedrae Herba | 22050411 | Caryophylli Flos | 22000321 |
| Eriobotryae Folium | 23010081 | Tribuli Fructus | 22028771 |
| Evodiae Fructus | 22038071 | Trichosanthis Radix | 22029491 |
| Longan Aril | 22036971 | Trichosanthis Semen | 22037861 |
| Foeniculi Fructus | 21023321 | Uncariae Uncis cum Ramulus | 22045681 |
| Forsythiae Fructus | 23012601 | Zanthoxyli Fructus | 22004261 |
| Fritillariae Bulbus | 23012111 | Zingiberis Processum Rhizoma | 23002281 |
| Gardeniae Fructus | 22031161 | Zingiberis Rhizoma | 22046221 |
| Gastrodiae Tuber | 23011631 | Zizyphi Frucuts | 22047121 |
| Gentianae Scubrae Radix | 22009991 | Zizyphi Spinosi Semen | 23006931 |
| Glehniae Radix cum Rhizoma | 22047301 | Poria | 23007381 |
| Glycyrrhizae Radix | 22053201 |  |  |
| All herbarium are purchased from Tsumura & Co. (Tokyo, Japan). | | | |

| Table S2. PAβN enhances effects of berberine in multidrug-resistant *P. aeruginosa* PAGU 1606. | | | | | |
| --- | --- | --- | --- | --- | --- |
|  |  | MIC (μg/ml) of  with (+) and without (-) addition of PAβN and BB^**^ | | | |
|  |  | AMK | | CIP | |
| Strain | Relevant property | - | + | - | + |
| PAGU 1606 | multidrug resistant | 256 | 64 | 128 | 8 |
| PAGU^g^1659 | PAGU 1606  Δ*XY* | 8 | 16 | 64 | 8 |
| ^**^: + or - indicates MICs in the absence or presence of both of PAβN (64 μg/ml) and/or BB (128 μg/ml).  PAβN MICs of both strains were >256 μg/ml.  Abbreviations: AMK, amikacin; CIP, ciprofloxacin; PAβN, phenylalanine-arginine β-naphthylamide; BB, berberine; *XY*, *mexXY* | | | | | |
