# Supplementary material for: Berberine Is a Novel Type Efflux Inhibitor Which Attenuates the MexXY-Mediated Aminoglycoside Resistance in Pseudomonas aeruginosa
Source: Front Microbiol. 2016 Aug 5;7:1223. doi: 10.3389/fmicb.2016.01223 (PMC4975076; doi:10.3389/fmicb.2016.01223)
Supplement: Supplementary file 2 [file Presentation1.PDF]

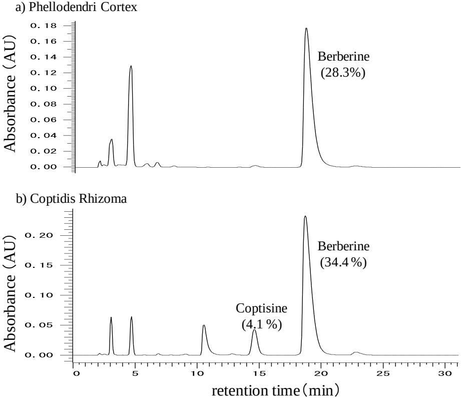

Fig. S1. Contents of berberine and coptisine in methanolic extracts from the rhizomes of *Coptis japonica* or the bark of *Phellodendron amurense*
